# A comparative analysis of performance and time-to-result for VITEK REVEAL versus MicroScan during antimicrobial susceptibility testing for gram-negative bacteremia

**DOI:** 10.1128/spectrum.01626-26

**Published:** 2026-09-02

**Authors:** Melissa Gitman, Albert Chow, Cecilia Hernandez, Manohar Mutnal, Noah Henry, Katelyn Teckenbrock, Shikha Chaudhary, Paul D. Fey, John S. Stevenson, Yolande A. Chan, Alexander D. Pyden, Matthew Kearney, Benjamin von Bredow, Amer Ait Chebib, Elizabeth M. Garrett, Meng Pu, Shivaramu Keelara, Richard Remington, Cecilia Carvalhaes, Carrie Lasky, Theresa Okeyo Owuor, April M. Bobenchik

**Affiliations:** 1 Icahn School of Medicine at Mount Sinai and Mount Sinai Health System 5925, New York, New York, USA; 2 Mount Sinai Health System 5944, New York, New York, USA; 3 Baylor Scott and White MC Temple, Temple, Texas, USA; 4 University of Nebraska Medical Center 12284, Omaha, Nebraska, USA; 5 Lahey Hospital and Medical Center 2094, Burlington, Massachusetts, USA; 6 Corewell Health 7005, Royal Oak, Michigan, USA; 7 Penn State Health Milton S. Hershey Medical Center, Hershey, Pennsylvania, USA; 8 bioMerieux, Inc., Durham, North Carolina, USA; Maryland Department of Health, Baltimore, Maryland, USA

**Keywords:** gram-negative bacteria, blood cultures, fast antimicrobial susceptibility testing, minimum inhibitory concentration, time to result, automated susceptibility testing, gram-negative bacilli, Enterobacterales

## Abstract

Fast and reliable antimicrobial susceptibility testing (AST) is essential for timely optimization of therapy in patients with bloodstream infections. This multicenter study assessed the performance of the bioMérieux VITEK REVEAL (VITEK REVEAL) system in comparison with the Beckman Coulter MicroScan (MicroScan) using positive blood culture (PBC) isolates. A total of 553 gram-negative PBCs were obtained from six clinical sites, with Enterobacterales representing the majority (91.1%, 504/553). Overall, 7,945 antimicrobial/organism combinations were evaluated to compare AST performance between VITEK REVEAL and MicroScan. VITEK REVEAL demonstrated strong concordance with MicroScan, achieving 98.1% essential agreement (EA) and 96.0% categorical agreement (CA). High agreement rates were observed for most antimicrobials, including amikacin, ceftazidime-avibactam, and meropenem-vaborbactam, each showing 100% EA and CA. Across all evaluations, discrepancies included 38 very major discrepancies (VMDs), 64 major discrepancies (MDs), and 218 minor discrepancies. Reference broth microdilution discrepant testing resolved most VMDs in favor of VITEK REVEAL, whereas most MDs aligned with MicroScan results. Species-level performance demonstrated strong agreement: *Escherichia coli* (98.1% EA, 95.6% CA), *Klebsiella pneumoniae* (99.2% EA, 97.7% CA), and *Pseudomonas aeruginosa* (97.4% EA, 95.1% CA). Time-to-result (TTR) analysis highlighted a major advantage of the VITEK REVEAL system. The mean overall TTR was 7.9 h (SD 0.4 h); sequential reporting enabled earlier results with a mean TTR of 6.5 h (SD 1.1 h). On average, VITEK REVEAL provided AST results 26–32 h (includes 18–24 h subculture for MicroScan) earlier than MicroScan, supporting its utility for fast, accurate AST reporting and potentially improving early antimicrobial decision-making.

**IMPORTANCE:**

This study represents one of the largest evaluations of VITEK REVEAL for fast antimicrobial susceptibility testing (AST) systems, including 553 gram-negative positive blood cultures across six clinical sites in the United States. The findings provide critical evidence for laboratories considering the adoption of fast AST, demonstrating that VITEK REVEAL delivers a strong combination of essential and categorical agreement (98.1% essential agreement [EA], 96.0% categorical agreement [CA] across 7,945 antimicrobial/organism combinations) and markedly reduced time-to-result (TTR). VITEK REVEAL performed consistently across key Enterobacterales and non-fermenters, including resistant phenotypes and β-lactam/β-lactamase inhibitor agents, underscoring its utility in diverse clinical scenarios. By providing susceptibility results 26–32 h earlier than MicroScan, this system may contribute to nimbler clinical decision-making, allowing for better antimicrobial stewardship practices.

## INTRODUCTION

The growing global burden of antimicrobial resistance (AMR) further intensifies the need for fast antimicrobial susceptibility testing (AST). The Global Research on Antimicrobial Resistance project reported 4.95 million deaths associated with bacterial AMR in 2019, including 1.27 million deaths directly attributable to AMR (1). Resistant pathogens, such as *Escherichia coli*, *Klebsiella pneumoniae*, and *Pseudomonas aeruginosa,* remain major contributors to this burden (2). In the United States, AMR remains a significant public health threat, despite robust surveillance and stewardship efforts. According to the U.S. Centers for Disease Control and Prevention (CDC) 2023 Special Report, more than 2.8 million antimicrobial-resistant infections occur annually, resulting in at least 35,000 deaths (3). Over the past decade, AMR in hospital-acquired infections has grown by 15%, driven by extended-spectrum β-lactamase (ESBL)-producing Enterobacterales, carbapenem-resistant *Acinetobacter*, and multidrug-resistant *Pseudomonas aeruginosa* (4).

AST is essential for guiding precise antimicrobial therapy, especially in life-threatening bloodstream infections and sepsis. Delays in AST reporting facilitate inappropriate empirical therapy, increased morbidity and mortality, and longer hospital stays (5). Conventional automated systems for AST, such as MicroScan, BD Phoenix, and VITEK 2, are widely used in clinical laboratories and require an additional 18–24 h for subculture from positive blood cultures (PBC) (6). These drawbacks limit their usefulness for early therapeutic decision-making, particularly in critical care settings. CDC emphasizes the need for new diagnostic approaches to improve AST, and implementation of advanced diagnostic platforms is a key component of its National Strategy for Combating Antibiotic-Resistant Bacteria 2020–2025 (7).

In this context, the VITEK REVEAL fast AST system (“VITEK REVEAL”), a recently FDA-cleared device (2024), is an AST system that provides susceptibility results directly from PBCs using metabolomic signature technology (8). The volatile organic compound-based metabolic detection technology of VITEK REVEAL enables earlier discrimination of growth patterns than conventional optical systems (9). Recent clinical studies have demonstrated significant reductions in AST time-to-result (TTR), with high categorical and essential agreement (CA and EA, respectively) between the VITEK REVEAL and comparator methodology (10–17). For example, Squitieri et al. (17) previously showed a twofold reduction in TTR (6.5 h vs 13.85 h) with VITEK REVEAL compared to the VITEK 2 direct-from-culture AST test, with no difference in accuracy (using broth microdilution as the reference method) (17).

Here, we present the results of a large, multicenter study, conducted to further investigate the analytical performance and TTR associated with VITEK REVEAL, compared to a conventional, automated AST (MicroScan WalkAway system) in the United States.

## MATERIALS AND METHODS

### Sample collection, organism ID, and AST

This was a prospective, non-interventional, multicenter (six clinical sites across the United States) study, conducted from July 2023 to January 2025. AST performance for the VITEK REVEAL system was compared with MicroScan at each site. Each site prospectively collected gram-negative PBCs. VITEK REVEAL was not used for diagnostic, treatment, or follow-up purposes, and the protocol was reviewed and approved by Institutional Review Boards at all sites. Specimens were included if they were positive blood cultures as detected using one of the following instruments, depending on site: BACT/ALERT 3D (bioMérieux SA; Marcy-l'Étoile, France) with FA/FN bottles; bioMérieux BACT/ALERT VIRTUO (bioMérieux SA; Marcy-l'Étoile, France) with FA Plus and/or FN Plus bottles; BD BACTEC (Becton, Dickinson and Company; Franklin Lakes, NJ) with Aerobic/F, Lytic/10 Anaerobic/F, or Peds Plus bottles; or BD BACTEC FX (Becton, Dickinson and Company; Franklin Lakes, NJ) system. In addition, a Gram stain was performed on each positive blood culture to confirm monomicrobial growth of gram-negative organisms. Fast organism identification was performed at each site using either the BioFire (bioMérieux SA; Marcy-l'Étoile, France) with the BCID 2 panel, the VERIGENE system (Diasorin SPA; Saluggia, Italy), the MALDI Biotyper with the MBT Sepsityper IVD kit (Bruker; Billerica, MA), or the cobas eplex system (Roche Diagnostics; Carlsbad, CA) with BCID panels. Samples were excluded if they lacked species identification or were not included in the VITEK REVEAL AST IFU; if AST on a specimen commenced more than 16 h after the positive blood culture was identified; they were polymicrobial; they were associated with system errors on either VITEK REVEAL (index test) or comparator system; or they were not gram-negative bacilli (e.g., they were yeast; gram-positive organisms).

PBCs were tested on the VITEK REVEAL AST System (bioMérieux SA; Marcy-l'Étoile, France), using the VITEK REVEAL GN BC-AST RUO panel, within 16 h of blood culture positivity (8).

Bacterial isolates from all PBCs were also tested on the MicroScan WalkAway system (“MicroScan”; Beckman Coulter, Brea, CA) using the NM56 panel, following the manufacturer’s instructions for use. If an invalid result was generated during VITEK REVEAL testing, the samples were reprocessed if they were still within the 16-h post-positivity window. All PBC isolates were stored in tryptic soy broth with 15% glycerol (or appropriate media) at ≤–70°C, using an 18–24 h culture for freezing. All VITEK REVEAL testing included quality control (QC) procedures as per the manufacturer’s instructions. Out-of-range QC results were repeated using freshly subcultured, pure strains.

### Outcomes and data analysis

EA for VITEK REVEAL was defined as the percentage of minimal inhibitory concentration (MIC) results within ±1 twofold dilution of the MicroScan result. CA was defined as the percentage of PBCs for which VITEK REVEAL and MicroScan yielded the same categorical interpretation—susceptible (S); susceptible-dose dependent (SDD); intermediate (I); or resistant (R). FDA STIC and CLSI M100 (34th edition) guidelines were applied for S/I/R categorization (18, 19). For cefepime testing with Enterobacterales, SDD (indicating expected efficacy only with defined dosing) was used in lieu of the intermediate interpretation, according to FDA STIC and CLSI M100 (34th edition) (18, 19). For piperacillin-tazobactam testing involving Enterobacterales, sites reported results as either I (FDA STIC) or SDD (CLSI M100; 34th edition); however, the same MIC breakpoint was used for both classifications. All sites verified their MicroScan reporting to be consistent with FDA STIC/CLSI M100 34th edition. Discrepancies were classified as very major discrepancy (VMD; resistant by MicroScan and susceptible by VITEK REVEAL), major discrepancy (MD; susceptible by MicroScan and resistant by VITEK REVEAL), and minor discrepancy (mD; discrepancies between S and I or between I and R), and were defined and measured as per Yusuf et al. (20). For ESBL screening, MIC values from cefotaxime alone and ceftazidime alone were compared to cefotaxime-clavulanate and ceftazidime-clavulanate, respectively. If clavulanate reduced the value of either cefotaxime alone or ceftazidime alone by ≥3 twofold dilutions, the screen indicated the presence of an ESBL-producing organism. Both VITEK REVEAL RUO and MicroScan NM56 use the same antibiotics and ≥3 doubling dilution difference to identify an ESBL. ESBL screening application was not included in the FDA-cleared version of the VITEK REVEAL panel. PBCs yielding VMD or MD between the VITEK REVEAL and MicroScan systems were sent to JMI Reference Laboratory (Element) for discrepancy resolution. Discrepant samples were tested in triplicate using reference broth microdilution (BMD). The modal MIC for BMD testing was interpreted into S/I/R, against which each sample associated with a VITEK REVEAL and MicroScan discrepancy was judged. PBCs with three distinct categorical BMD results (S, I, R) were excluded.

TTR was compared between VITEK REVEAL and MicroScan. Final TTR results reporting reflected the time for all results to become available across the full antimicrobial panel. Sequential reporting involved a stewardship-driven approach in which results by individual antimicrobials were obtained in a stepwise manner prior to final results reporting. TTR was recorded in hours (h) for each antimicrobial/organism combination. TTR was calculated by determining the sum of hours of instrument run time for VITEK REVEAL or instrument run time for MicroScan (which included an additional 18–24 h time for subculture). The original data corresponding to this study can be found on FigShare (DOI: 10.6084/m9.figshare.33233700) (21).

## RESULTS

A total of 553 samples were collected directly from PBCs across six sites. Overall, across 7,945 antimicrobial/organism combinations, VITEK REVEAL showed high EA (98.1%) and CA (96.0%) when compared to MicroScan AST results. Of the 772 resistant AST results by MicroScan, 38 (4.9%) were susceptible (VMD) by VITEK REVEAL, and of the 6,994 susceptible MicroScan results, 64 (1.0%) corresponded to resistant (MD) VITEK REVEAL results (Table 1). The remaining 2.7% mismatch between VITEK REVEAL and MicroScan occurred due to minor discrepancies (218/7,945).

**TABLE 1:** Overall antimicrobial susceptibility testing performance of VITEK REVEAL compared to MicroScan

Agreement and discrepancies	% (*n*/total)
Essential agreement	98.1% (7,792/7,945)
Categorical agreement	96.0% (7,625/7,945)
Very major discrepancy	4.9% (38/772)
Major discrepancy	1.0% (64/6,994)
Minor discrepancy	2.7% (218/7,945)

Enterobacterales accounted for 91.1% (504/553) of isolates, including *E. coli* (52.4%; 290/553), *K. pneumoniae* group (19.3%; 107/553), and a combined (19.3%; 107/553) from *Enterobacter cloacae* complex, *Proteus mirabilis*, *Serratia marcescens*, *Klebsiella aerogenes*, *Klebsiella oxytoca*, and *Citrobacter* spp. (Table 2). Non-Enterobacterales accounted for the remaining isolates, with *P. aeruginosa* (45/553) and *Acinetobacter baumannii* complex (4/553) accounting for 8.9% of total specimens. The number of isolates displaying S/I/R results by species and by antimicrobial tested are displayed in the supplementary material (Tables S1 and S2, respectively).

**TABLE 2:** Antimicrobial susceptibility testing results for VITEK REVEAL as compared to MicroScan reported by organism^*a*^^,^^*d*^^,^^*e*^

Organism	Strains (*n*)	Antimicrobials (*n*)	Strains-antimicrobials^*b*^	% EA (*n*)	% CA (*n*)	VMD (%)	MD (%)	mD (%)
*Citrobacter freundii*	4	8	26	100 (26)	96.2 (25)	0.0 (0/3)	0.0 (0/22)	3.85 (1/26)
*Citrobacter koseri*	4	9	35	100 (35)	100 (35)	–^*f*^	0.0 (0/35)	0.0 (0/35)
*E. cloacae* complex	20	13	200	94.0 (188)	92.5 (185)	17.4 (4/23)	2.3 (4/172)	3.5 (7/200)
*E. coli*	290	19	4,752	98.1 (4,660)	95.6 (4,544)	3.6 (19/532)	1.1 (44/4,092)	3.1 (145/4,752)
*K. aerogenes*	10	10	94	96.8 (91)	94.7 (89)	37.5 (3/8)	0.0 (0/84)	2.1 (2/94)
*K. oxytoca*	20	13	233	98.3 (229)	98.3 (229)	9.1 (1/11)	1.4 (3/220)	0.0 (0/223)
*K. pneumoniae* group	107	17	1,688	99.2 (1,675)	97.7 (1,649)	3.6 (5/141)	0.3 (5/1,528)	1.7 (29/1,688)
*P. mirabilis*	34	12	349	96.0 (335)	94.3 (329)	3.7 (1/27)	2.5 (8/317)	3.2 (11/349)
*S. marcescens*	15	7	90	98.9 (89)	95.6 (86)	0.0 (0/1)	0.0 (0/84)	4.4 (4/90)
**Enterobacterales** ^*c*^	**504**	**19**	**7,467**	**98.1 (7,328)**	**96.0 (7,171)**	**4.4 (33/756)**	**1.0 (64/6,554)**	**2.7 (199/7,467)**
***A. baumannii*** **complex**	**4**	**4**	**13**	**92.3 (12)**	**92.3 (12)**	–	**0.0 (0/13)**	**7.7 (1/13)**
***P. aeruginosa***	**45**	**11**	**465**	**97.4 (453)**	**95.1 (442)**	**19.2 (5/26)**	**0.0 (0/427)**	**3.9 (18/465)**

^*a*^ Current VITEK REVEAL and MicroScan limitations are applied to the analysis.

^*b*^ Number for pairwise comparisons.

^*c*^ Overall Enterobacterales.

^*d*^ EA, essential agreement; CA, categorical agreement; VMD, very major discrepancy; MD, major discrepancy; mD, minor discrepancies.

^*e*^ Boldface indicates the organism groups being compared.

^*f*^ –, no data.

Overall, VITEK REVEAL EA and CA for Enterobacterales were 98.1% and 96.0%, respectively. Species-level analysis showed EA ≥ 96.0% for all organisms except *E. cloacae* complex (94.0%; 188/200). The overall CA for Enterobacterales was 96.0% (7,171/7,467). *E. coli* and *K. pneumoniae* group were associated with a CA of 95.6% (185/200) and 97.7% (1,649/1,688), respectively. The lowest CA was associated with *E. cloacae* complex (92.5%; 185/200). Discrepancy rates varied across organisms and antimicrobial categories. Overall, the VMD rate for Enterobacterales was 4.4%. VMD for both *E. coli* and *K. pneumonia* was 3.6%. The organisms corresponding with the largest VMD values were *K. aerogenes* (37.5%; 3/8), *E. cloacae* complex (17.4%; 4/23), and *K. oxytoca* (9.1%; 1/11). Across all organisms, MD rates were low (≤2.5%).

Among non-fermenting gram-negative bacteria, *P. aeruginosa* demonstrated EA and CA values of 97.4% and 95.1%, respectively, while *A. baumannii* complex showed EA and CA values of 92.3% and 92.3%, respectively (Table 2). A total of five (19.2%) VMDs were observed for *P. aeruginosa*, including two aztreonam results and one of each: cefepime, ciprofloxacin, and levofloxacin (Table S3).

Across the 19 antimicrobials evaluated, VITEK REVEAL showed high overall concordance with MicroScan (Table 3). EA exceeded 95% for all agents and reached 100% for amikacin, ceftazidime/avibactam, and meropenem/vaborbactam. CA remained ≥92.0% for all antimicrobials except ampicillin/sulbactam (85.2%; 325/338). CA was 100% for amikacin (*n* = 407), ceftazidime/avibactam (*n* = 392), and meropenem/vaborbactam (*n* = 327).

**TABLE 3:** Antimicrobial susceptibility testing results for VITEK REVEAL as compared to MicroScan reported by antimicrobial^*e*^

Antimicrobial	Strains	% EA	% CA	VMD (%)	MD (%)	mD (%)
Amikacin^*a*^	407	100 (407/407)	100 (407/407)	0.00 (0/1)	0.0 (0/361)	0.0 (0/363)
Amoxicillin-clavulanate^*b*^	415	98.6 (409)	93.0 (386)	10.7 (3/28)	0.8 (3/363)	5.5 (23/415)
Ampicillin-sulbactam	338	96.2 (325)	85.2 (288)	1.6 (1/61)	2.9 (6/208)	12.7 (43/338)
Aztreonam^*a*^	424	96.9 (411)	94.6 (401)	6.4 (5/78)	2.0 (6/296)	1.3 (5/379)
Cefepime^*c*^	495	95.4 (472)	94.7 (469)	2.5 (2/80)	1.1 (4/366)	4.2 (19/450)
Ceftazidime	453	96.9 (439)	93.8 (425)	6.9 (5/72)	1.9 (7/362)	3.3 (15/449)
Ceftazidime-avibactam	392	100 (392)	100 (392)	0.0 (0/2)	0.0 (0/348)	0.0 (0/350)
Ceftolozane-tazobactam	315	98.1 (309)	97.1 (306)	25.0 (2/8)	1.5 (4/262)	1.1 (3/272)
Ceftriaxone	453	96.2 (436)	96.0 (435)	3.9 (3/78)	2.2 (8/370)	1.6 (7/453)
Ciprofloxacin	447	97.5 (436)	92.8 (415)	2.3 (2/89)	2.3 (7/300)	4.5 (18/404)
Ertapenem	348	99.4 (346)	98.6 (343)	0.0 (0/5)	0.6 (2/343)	0.9 (3/348)
Gentamicin*^a^*^,*b*^	524	98.7 (517)	98.1 (514)	1.9 (1/52)	0.5 (2/427)	1.0 (5/481)
Imipenem^*b*^	341	98.8 (337)	96.2 (328)	0.0 (0/2)	0.3 (1/302)	2.9 (9/309)
Levofloxacin^*b*^	522	98.7 (515)	95.4 (498)	3.2 (3/93)	0.5 (2/380)	2.9 (14/477)
Meropenem	512	99.6 (510)	99.2 (508)	0.0 (0/3)	0.4 (2/458)	0.4 (2/463)
Meropenem-vaborbactam	327	100 (327)	100 (327)	0.0 (0/2)	0.0 (0/325)	0.0 (0/327)
Piperacillin-tazobactam*^b^*^,*d*^	387	96.4 (373)	93.0 (360)	19.1 (4/21)	1.1 (4/359)	4.9 (19/387)
Tobramycin^*b*^	527	97.7 (515)	96.6 (509)	0.0 (0/43)	0.9 (4/434)	3.0 (14/484)
Trimethoprim-sulfamethoxazole^*b*^	318	99.7 (317)	98.7 (314)	7.1 (2/28)	0.7 (2/290)	0.0 (0/318)

^*a*^ Antibiotics present in the GN BC-AST (RUO) panel used for this study but are not included on the GN02 commercial panel.

^*b*^ Results exclude reporting limitations for some organism/antibiotic combinations with specific MICs that require confirmatory testing with an alternate method as indicated in the IVD package insert.

^*c*^ For Enterobacterales, the susceptible-does dependent classification was used instead of intermediate.

^*d*^ For Enterobacterales, the either intermediate or susceptible-does dependent classifications were used, depending on the site; minimum inhibitory concentration was the same for both classifications.

^*e*^ EA, essential agreement; CA, categorical agreement; VMD, very major discrepancy; MD, major discrepancy; mD, minor discrepancies.

The majority (11/19) of antimicrobials were associated with VMD rates ≤3.0%. VMD above 3.0% was observed for amoxicillin-clavulanate (10.7%; 3/28), piperacillin-tazobactam (19.1%; 4/21), ceftolozane-tazobactam (25.0%; 2/8), trimethoprim-sulfamethoxazole (7.1%; 2/28), ceftazidime (6.9%; 5/72), aztreonam (6.4%; 5/78), ceftriaxone (3.9%; 3/78), and levofloxacin (3.2%; 3/93). MDs remained below 3% for all antimicrobials, and mDs were generally ≤10% except ampicillin-sulbactam (12.7%; 43/338). Carbapenems (imipenem, meropenem, ertapenem) and the new β-lactam/β-lactamase inhibitor combinations showed particularly strong agreement profiles (Table 3).

ESBL-positive strains were identified and recorded by five of the six clinical sites by both MicroScan, and the VITEK Reveal ESBL screen test. For screening for ESBL-producing organisms, VITEK REVEAL showed a CA of 95.2% when compared to MicroScan results for all organisms tested (Table S4).

Among the 38 VMDs for VITEK REVEAL, BMD resolved 22 (57.9%) in favor of VITEK REVEAL and seven (18.4%) in favor of MicroScan, while nine (23.7%) were not resolved. For the 64 MDs associated with VITEK REVEAL compared to MicroScan, 14 (21.9%) were resolved in favor of VITEK REVEAL and 43 (67.2%) in favor of MicroScan; six (9.4%) were unresolved (Table 4). Overall VMD and MD rates for VITEK REVEAL compared to MicroScan, following discrepant resolution with BMD, were 2.1% (16/772) and 0.7% (50/6,994), respectively (Table S5).

**TABLE 4:** Comparison of discrepancies between VITEK REVEAL and MicroScan and resolution by broth microdilution^*a*^

	All discrepancies (*n*)				Very major discrepancies (*n*)				Major discrepancies (*n*)			
		Resolved by BMD				Resolved by BMD				Resolved by BMD		
Antimicrobials	REVEAL vs MicroScan	REVEAL	MicroScan	Not resolved by BMD	REVEAL vs MicroScan	REVEAL	MicroScan	Not resolved by BMD	REVEAL vs MicroScan	REVEAL	MicroScan	Not resolved by BMD
Amoxicillin/clavulanate	6	3	3	0	3	3	0	0	3	0	3	0
Ampicillin/sulbactam	7	0	7	0	1	0	1	0	6	0	6	0
Aztreonam	13	4	2	7	7	3	0	4	6	1	2	3
Cefepime	7	1	4	2	3	0	1	2	4	1	3	0
Ceftazidime	12	7	4	1	5	4	1	0	7	3	3	1
Ceftolozane/tazobactam	6	0	6	0	2	0	2	0	4	0	4	0
Ceftriaxone	11	6	5	0	3	3	0	0	8	3	5	0
Ciprofloxacin	10	5	4	1	3	2	0	1	7	3	4	0
Ertapenem	2	0	0	2	0	0	0	0	2	0	0	2
Gentamicin	3	1	2	0	1	1	0	0	2	0	1	0
Imipenem	1	0	1	0	0	0	0	0	1	0	1	0
Levofloxacin	6	3	1	2	4	2	0	2	2	1	1	0
Meropenem	2	0	2	0	0	0	0	0	2	0	2	0
Piperacillin/tazobactam	8	4	4	0	4	2	2	0	4	2	2	0
Tobramycin	4	0	4	0	0	0	0	0	4	0	4	0
Trimethoprim/sulfamethoxazole	4	2	2	2	2	2	0	0	2	0	2	0
Overall (*n*)	102	36	51	17	38	22	7	9	64	14	43	6
Overall (%)		35.3	50.0	16.7		57.9	18.4	23.7		21.9	67.2	9.4

^*a*^ BMD, broth microdilution.

**Fig 1:**
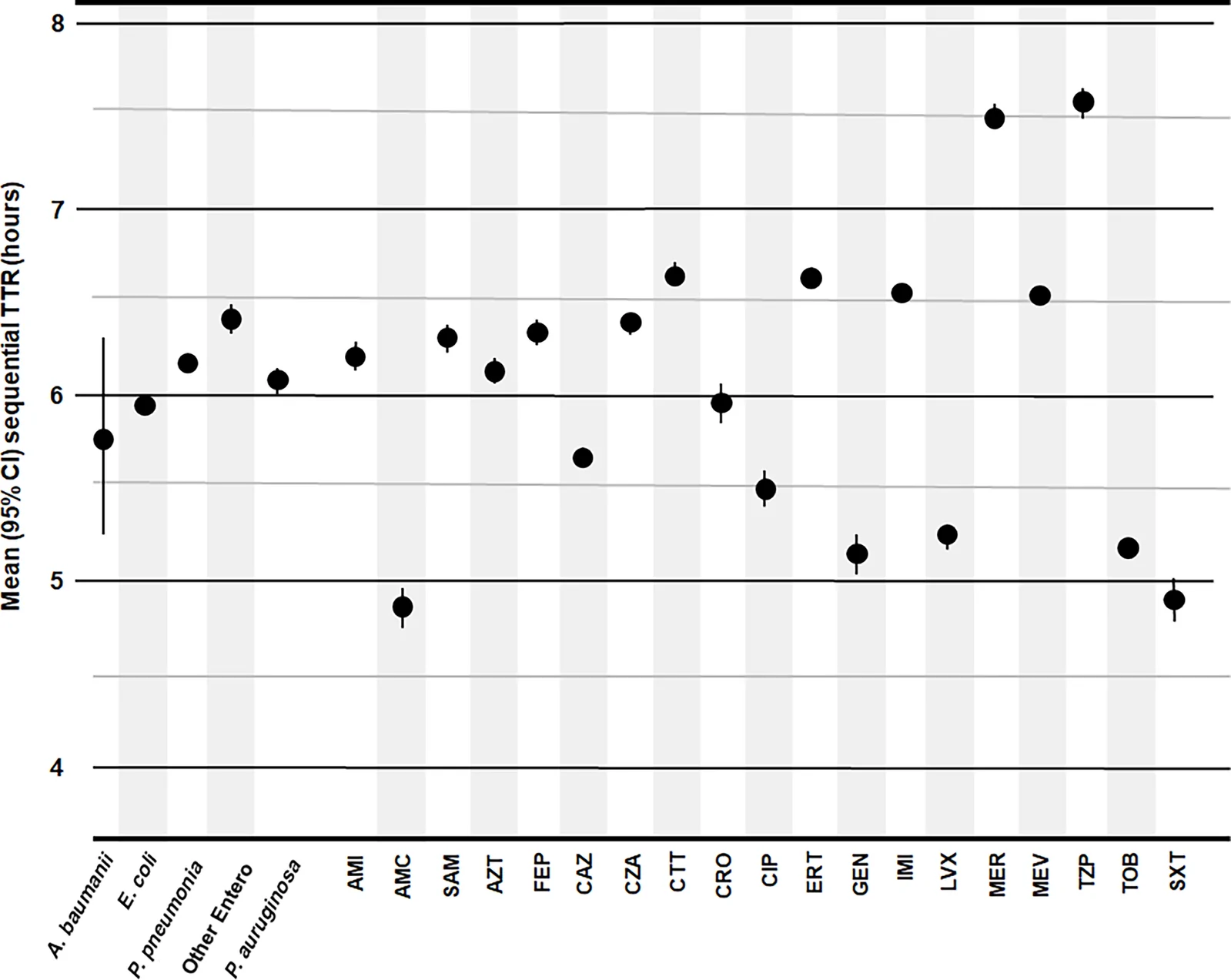
Time-to-result during AST across species and antibiotics. Mean values and associated 95% confidence intervals are shown in black circles and vertical lines, respectively. AMI, amikacin; AMC, amoxicillin-clavulanate; SAM, ampicillin-sulbactam; AZT, aztreonam; FEP, cefepime; CAZ, ceftazidime; CZA, ceftazidime-avibactam; CTT, ceftolozane-tazobactam; CRO, ceftriaxone; CIP, ciprofloxacin; ERT, ertapenem; GEN, gentamicin; IMI, imipenem; LVX, levofloxacin; MER, meropenem; MEV, meropenem-vaborbactam; TZP, piperacillin-tazobactam; TOB, tobramycin; SXT, trimethoprim-sulfamethoxazole.

Across all organism groups (Table 5), VITEK REVEAL demonstrated substantially shorter TTR values compared to MicroScan. Regardless of the organism group/species or antimicrobial agent, MicroScan TTR was 34–40 h (16 h instrument run time plus 18–24 h for subculture). The median final VITEK REVEAL TTR for Enterobacterales and non-fermenters was 8.0 and 6.5 h, respectively. Overall, sequential VITEK REVEAL results were available earlier (median TTR of 6.5 h and mean TTR of 6.0 h). *E. coli* (290 PBCs tested) showed a mean TTR of 5.9 h; the other Enterobacterales group showed a mean sequential TTR of ~6.4 h. *A. baumannii* complex was associated with a lower mean TTR, but only four *A. baumannii* complex PBCs were included in this study (Fig. 1). Sequential TTRs by antimicrobial agent are shown in Table 6. Although a few antimicrobial categories showed sequential TTRs above 7 h (meropenem and piperacillin-tazobactam), most antimicrobial category sequential TTRs were below 7 h. Six antimicrobials were associated with sequential TTRs below 6 h; these included amoxicillin/clavulanate, ceftazidime, ciprofloxacin, gentamicin, levofloxacin, tobramycin, and trimethoprim/sulfamethoxazole.

**TABLE 5:** VITEK REVEAL time-to-result values according to organism category^*a*^

		REVEAL final TTR (h)			REVEAL sequential TTR (h)		
Organism	Number	Median	Range	Mean (SD)	Median	Range	Mean (SD)
*E. coli*	290	8.0	(6.5, 9.1)	8.0 (0.1)	6.3	(3.0, 8.2)	5.9 (1.2)
*K. pneumoniae*	107	8.0	(6.5, 8.1)	8 (0.3)	6.5	(3.0, 7.0)	6.2 (0.7)
Other Enterobacterales	107	8.0	(7, 8.2)	8.0 (0.1)	6.5	(3.2, 8.2)	6.4 (1.2)
*A. baumannii* complex	4	6.5	(6.5, 6.5)	6.5 (0)	6.0	(4.5, 6.5)	5.7 (0.9)
*P. aeruginosa*	45	6.5	(6.5, 6.6)	6.5 (0.1)	6.5	(4.0, 6.6)	6.1 (0.8)
Overall	553	8.0	(6.5, 9.1)	7.9 (0.4)	6.5	(3.0, 8.2)	6.0 (1.1)

^*a*^ TTR, time-to-result.

**TABLE 6:** VITEK REVEAL time-to-result values according to antimicrobial category^*a*^

		REVEAL final TTR (h)			REVEAL sequential TTR (h)		
Organism	Number	Median	Range	Mean (SD)	Median	Range	Mean (SD)
Amikacin	407	8	(6.5, 8.2)	7.9 (0.5)	6.5	(4, 8.1)	6.2 (0.8)
Amoxicillin/clavulanate	415	8	(6.5, 9.1)	8.0 (0.2)	4.3	(3.1, 6.7)	4.9 (1.0)
Ampicillin/sulbactam	338	8	(6.5, 9.1)	8.0 (0.2)	6.5	(3.2, 6.7)	6.3 (0.7)
Aztreonam	425	8	(6.5, 8.2)	7.9 (0.5)	6.1	(3, 8.2)	6.1 (0.7)
Cefepime	496	8	(6.5, 9.1)	7.9 (0.5)	6.5	(3.2, 8.0)	6.3 (0.7)
Ceftazidime	454	8	(6.5, 9.1)	8.0 (0.2)	5.5	(3.1, 6.7)	5.7 (0.5)
Ceftazidime/avibactam	392	8	(6.5, 8.2)	8.0 (0.5)	6.5	(5.7, 6.7)	6.4 (0.2)
Ceftolozane/tazobactam	315	8	(6.5, 8.2)	7.2 (0.5)	6.5	(3.3, 8.1)	6.7 (0.5)
Ceftriaxone	453	8	(6.5, 9.1)	8.0 (0.2)	6.0	(3, 8.1)	6.0 (1.1)
Ciprofloxacin	448	8	(6.5, 8.2)	7.9 (0.5)	5.5	(3, 8.1)	5.5 (1.0)
Ertapenem	348	8	(6.5, 8.2)	8.0 (0.2)	6.5	(3.1, 8.1)	6.6 (0.5)
Gentamicin	524	8	(6.5, 9.1)	7.9 (0.4)	4.3	(3.2, 8.1)	5.2 (1.2)
Imipenem	342	8	(6.5, 8.2)	7.9 (0.5)	6.5	(6.5, 8.0)	6.6 (0.2)
Levofloxacin	523	8	(6.5, 9.1)	7.9 (0.5)	5.0	(4.0, 8.1)	5.6 (0.8)
Meropenem	513	8	(6.5, 9.1)	8.0 (0.5)	8.0	(3.1, 8.2)	7.5 (0.8)
Meropenem/vaborbactam	328	8	(6.5, 8.2)	8.0 (0.1)	6.5	(6.5, 6.7)	6.5 (0.1)
Piperacillin/tazobactam	387	8	(6.5, 9.1)	8.0 (0.2)	8.0	(3.1, 8.2)	7.6 (0.8)
Tobramycin	528	8	(6.5, 9.1)	7.9 (0.4)	5.0	(3.3, 8.1)	5.2 (0.7)
Trimethoprim/sulfamethoxazole	318	8	(6.5, 8.2)	8.0 (0.2)	4.2	(4, 6.7)	4.9 (1.1)

^*a*^ TTR, time-to-result.

**Fig 2:**
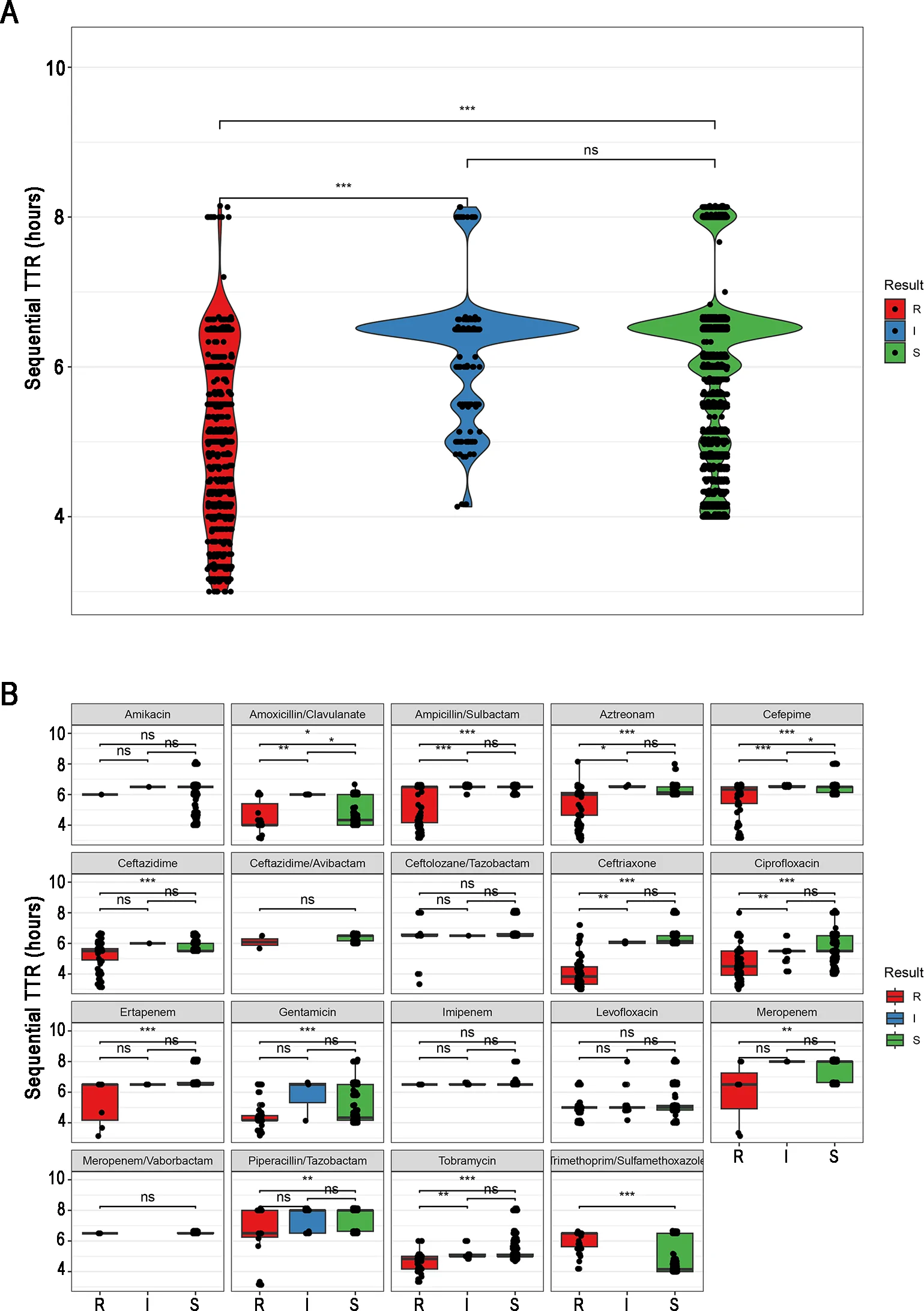
Distribution of times to AST results across antimicrobials used during testing for resistant and susceptible strains. (**A**) Violin plot showing TTR (hours) values for 7,945 antimicrobial-organism pairs, grouped by susceptibility (S, I, R). Each dot represents one organism-antimicrobial combination. Violin width reflects data density. (**B**) Box plots of the same data, grouped by susceptibility and antimicrobial. Boxes show the interquartile range; whiskers span the full range; black lines denote the median. TTR (y-axis) is in hours. S = susceptible; I = intermediate; R = resistant; ns = not significant. Points near 8 h in panel **A** reflect the assay’s maximum runtime. For both panels, asterisks denote significant differences (S vs R, S vs I, I vs R); *P < 0.05; **P < 0.01; ***P < 0.001; ****P < 0.0001; ns, not significant.

Figure 2 demonstrates TTR associated with susceptible, intermediate, and resistant organisms across antimicrobials. Overall, the distribution of TTR values for resistant PBCs was lower than those for intermediate or susceptible categories (Fig. 2A). However, several antimicrobial categories were associated with high variability among the resistant isolates. These included ceftazidime-avibactam, ertapenem, meropenem, and piperacillin-tazobactam (Fig. 2B).

## DISCUSSION

This multicenter study aimed to evaluate the analytical performance and TTR for the VITEK REVEAL fast AST system as compared to MicroScan. Across all organisms tested, VITEK REVEAL demonstrated excellent overall concordance with MicroScan, achieving 98.1% EA and 96.0% CA. Although the overall VMD rate was >3% (4.9%), resolution of discordant VITEK REVEAL results with BMD resulted in a VMD rate that was <3% (2.1%), which is considered an acceptable rate according to CLSI recommendations (22). TTR analysis demonstrated that the VITEK REVEAL system delivers fast antimicrobial susceptibility results across a wide range of agents. The system consistently produced results within a median of 6.5–8 h, shorter than the fixed 34–40 h required by MicroScan. Sequential VITEK REVEAL outputs provided even earlier actionable interpretations (mean of 6.0 h). Finally, ESBL screening, using VITEK REVEAL, showed high agreement (>95%) with the MicroScan ESBL screen. This could enable timely detection of resistant organisms and improve patient management by guiding clinicians toward effective therapy. Although early identification of ESBL-producing organisms can promote antimicrobial stewardship and infection control efforts, ESBL screening on the currently marketed VITEK REVEAL GN panel is not currently available.

Several studies, including five from Italy (9–11, 14, 17), three from the United States (12, 16, 23), and one each from France (13), Germany (15), and South Korea (24), reported AST results for VITEK REVEAL. Of the 11 studies, two compared VITEK REVEAL directly to MicroScan, five compared to BMD, and the remaining studies compared to other commercial AST devices (Table S6). Overall, CA for the 11 studies listed in Table S6 ranged from 94% to 99%. Available EA values from those studies ranged from 96% to 100%. Consistent with previous work (11, 14, 17), in this study, VITEK REVEAL showed high EA and CA for Enterobacterales, including *E. coli*, *K. pneumoniae*, and *Citrobacter* spp., as well as the non-fermenters *P. aeruginosa* and *A. baumannii* complex (10, 11, 13, 14, 17). For overall VMD, seven of the previously mentioned studies reported overall rates below 3% (10, 11, 13, 14, 16, 17, 24); four studies reported rates above 3% (ranging from 3.6% to 16.1%) (9, 12, 15, 23). Of the six studies reporting on Enterobacterales (11, 14–17, 23), four (11, 14, 16, 17) included a VMD rate <3%; of the seven studies reporting on non-fermenters (10, 11, 13–15, 17, 23), six included a VMD rate <3% (10, 11, 13–15, 17).

Three published studies have reported VITEK REVEAL analytical performance and TTR in the United States. Across the three studies, Tibbetts et al. (16), Bui et al. (12), and Simmons-Williams et al. (23) were each conducted at single large academic hospital systems, Henry Ford Health System (Detroit), Barnes Jewish Hospital (St. Louis), and Tampa General Hospital (Tampa), respectively, and collectively evaluated over 350 positive blood cultures with more than 5,000 antimicrobial/organism combinations, including 128 PBCs and 1,410 combinations in the Simmons-Williams study alone (12, 16, 23). The study described here was a multicenter study conducted across six sites in the United States that included 553 isolates and 7,945 antimicrobial/organism combinations. In this study, the overall CA was very close to the previous three US-based studies (96.0% vs 94.3%, 94.8%, and 96.2% in references 12, 16, 23, 12, and 16, respectively); Bui et al. did not report EA (due to disk diffusion as the reference), but similar overall EA results were obtained between this study (98.1%) compared to those reported by Simmons et al. (96.0%) and Tibbetts et al. (97.0%). Although Bui et al. did not report TTR, the overall sequential TTR for Simmons-Williams et al. (6.1 h) and Tibbetts et al. (6.2 h) was close to that reported here (6.0 h).

Although the number of VMDs remained low overall, in this study, certain species were associated with relatively high VMD rates. These included *K. aerogenes, P. aeruginosa*, *E. cloacae*, and *K. oxytoca*. Tibbetts et al. (16) also reported high VMD rates associated with *K. oxytoca* and *E. cloacae*. In that study, VITEK 2, another automated AST system, was used as the comparator test. However, the number of resistant (according to the comparator) *K. oxytoca* and *E. cloacae* isolates corresponding to these organisms, from both Tibbetts et al. and those reported here, was low. MicroScan reported 26 resistant *P. aeruginosa* PBCs (including seven strains to levofloxacin, six each to aztreonam and ciprofloxacin, five to cefepime, and one each to imipenem and meropenem), of which VITEK REVEAL called five as susceptible (VMD rate of 19%); this is higher than that reported in previous studies (range 0.0% to 6.2%) (11, 13–15, 17). Discrepant analysis of the five PBCs with BMD as the reference reported one isolate as susceptible, one as resistant, and two as intermediate; one isolate was not tested (data not shown); thus, the post-discrepant reconciliation VMD rate was 15.4%.

Nineteen (19) antimicrobial agents were utilized for testing across the 553 isolates included in this study. No VMDs were observed for ceftazidime-avibactam, ertapenem, meropenem-vaborbactam, or imipenem, which are often reserved for multidrug-resistant organisms. These results parallel earlier findings in which VITEK REVEAL demonstrated reliable carbapenem and β-lactam/β-lactamase inhibitor performance (15, 17). However, VITEK REVEAL showed VMD rates of ≥3% for amoxicillin-clavulanate, aztreonam, ceftazidime, ceftolozane-tazobactam, ceftriaxone, levofloxacin, piperacillin-tazobactam, and trimethoprim-sulfamethoxazole. VMD values of ≥3% have been reported previously, including two reports for amoxicillin-clavulanate (12, 15), two for aztreonam (12, 17), four for ceftazidime (12, 13, 15, 24), two for ceftolozane-tazobactam (12, 13), and three for piperacillin-tazobactam (11, 12, 15). Several factors could account for the differences in reported VMDs associated with specific antimicrobials. These include the region or country in which the studies were conducted, the reference test utilized for performance evaluation, study design (for example, utilization of a high prevalence of resistant isolates as opposed to a random collection protocol), and the breakpoint guidelines used in the study.

Here, VITEK REVEAL led to a significant reduction in TTR for AST results. Achieving TTRs of ≤6.0 h for antibiotics such as ciprofloxacin and ceftriaxone may offer meaningful clinical utility by shortening the time to targeted therapy in bloodstream infections (25). These data align with previous reports demonstrating accelerated TTRs for VITEK REVEAL, including median times of 3.6–7.3 h reported by Ostermann et al. (15) and studies comparing VITEK REVEAL with VITEK 2 or fast AST methodologies (15–17, 26). The reduction in TTR by 26–32 h with VITEK REVEAL, noted here, is clinically meaningful as earlier AST results can expedite targeted therapy, support antimicrobial de-escalation, and improve outcomes in bloodstream infections (17, 27–29). This workflow advantage underscores VITEK REVEAL’s substantial impact, providing actionable susceptibility information more than a full day sooner than MicroScan.

Reductions in TTR are associated with shorter times to appropriate or definitive antimicrobial therapy, including earlier de-escalation (30, 31). By reducing diagnostic uncertainty, fast AST supports antimicrobial stewardship goals by limiting unnecessary broad-spectrum exposure, thereby mitigating antimicrobial resistance, drug-related toxicity, and other downstream adverse outcomes (28, 29). Faster availability of susceptibility results may improve stewardship efforts, particularly in settings with high prevalence of ESBL-producing Enterobacterales, carbapenem-resistant *Acinetobacter* spp., and multidrug-resistant *P. aeruginosa*. Taken together, even in the absence of directly measured prescribing endpoints, improvements in TTR represent a clinically meaningful advance, as they provide the foundation necessary to facilitate earlier escalation and de-escalation decisions in routine clinical practice. Additionally, VITEK REVEAL’s consistent performance across antimicrobial agents—including aminoglycosides, third-generation cephalosporins, and novel β-lactam/β-lactamase inhibitor combinations—supports its applicability in diverse clinical scenarios.

The inverse relationship between TTR and antimicrobial resistance has been demonstrated in several studies. Results from this study further confirm that resistant phenotypes are identified faster than non-resistant phenotypes when using VITEK REVEAL. Ostermann et al. emphasized this as a diagnostic advantage for VITEK REVEAL and other fast AST systems (15). Timely administration of appropriate antimicrobial treatment is critical for addressing bloodstream infections caused by difficult-to-treat resistance organisms (29). TTR results for resistant isolates were significantly lower for both first-line antimicrobial agents, such as ampicillin-sulbactam, cefepime, ceftazidime, and ciprofloxacin, and second-line antimicrobials, including ertapenem and tobramycin.

### Strengths and limitations

This study offers several strengths, including its multicenter design, large and diverse isolate collection, head-to-head comparison with a conventional, automated AST system, and systematic discrepancy resolution using triplicate BMD. By incorporating multiple clinical settings, the current results strengthen the evidence base for VITEK REVEAL fast AST accuracy and TTR in the United States and provide a more comprehensive view of real-world implementation of fast AST for the microbiology workflow.

Certain limitations exist regarding this study. Broth microdilution was performed only for isolates showing discrepant results between VITEK REVEAL and MicroScan. As a result, any platform-dependent errors that were concordant across both systems would not have been detected. This may have led to an underestimation of true error rates and limits the ability to fully assess the accuracy of each method relative to the reference standard. Additionally, some resistance phenotypes were represented in limited numbers, reducing the statistical power to evaluate performance in these subgroups.

Although our study was not designed to evaluate clinical outcomes, the observed reduction in AST TTR may have important implications for antimicrobial optimization. Prior studies suggest that faster phenotypic AST can improve process-of-care measures in bloodstream infections, including earlier antimicrobial escalation or de-escalation, and shorter time to optimized therapy (32–34). For example, Oudiane et al. reported that implementation of fast AST increased the proportion of patients receiving optimized therapy within 24 h of blood culture positivity and reduced time to optimized therapy by nearly 20 h (34). Similarly, observational studies of rapid phenotypic AST, including VITEK REVEAL, have suggested associations with earlier effective therapy and improved clinical outcomes, although these findings remain limited by non-randomized designs and potential residual confounding (33, 35). Our findings are consistent with this literature by demonstrating shorter time to actionable AST information. However, the clinical impact of fast AST likely depends on baseline empiric therapy adequacy, illness severity, antimicrobial stewardship infrastructure, and clinician response to results (32, 36). Further prospective studies are needed to determine whether reduced time to results translates into consistent improvements in mortality, length of stay, antimicrobial use, or other patient-centered outcomes.

### Conclusion

Collectively, the findings demonstrate that the VITEK REVEAL system provides high concordance, fast sequential reporting, and direct-from-positive blood culture workflow, representing a significant advance over conventional automated AST. Early reporting potentially enhances its clinical utility by enabling faster therapeutic decision-making. Future work should assess the impact of VITEK REVEAL-guided therapy on clinical outcomes, evaluate performance across broader resistance phenotypes, and explore integrating fast identification and fast AST within real-world stewardship programs.

## SUPPLEMENTAL MATERIAL

Supplemental tables spectrum.01626-26-s0001.docx Tables S1 to S6.

ASM does not own the copyrights to Supplemental Material that may be linked to, or accessed through, an article. The authors have granted ASM a non-exclusive, world-wide license to publish the Supplemental Material files. Please contact the corresponding author directly for reuse.
