# Supplementary material for: A comparative analysis of performance and time-to-result for VITEK REVEAL versus MicroScan during antimicrobial susceptibility testing for gram-negative bacteremia
Source: Microbiol Spectr. 2026 Sep 2;14(10):e01626-26. doi: 10.1128/spectrum.01626-26 (PMC13689412; doi:10.1128/spectrum.01626-26)
Supplement: Supplementary file 1 [file spectrum.01626-26-s0001.docx]

# **SUPPLEMENTAL MATERIAL**

A comparative analysis of performance and time-to-result for VITEK REVEAL versus MicroScan during antimicrobial susceptibility testing for gram-negative bacteremia

Melissa Gitman, MD, MPH,^1^ Albert Chow,^2^ Cecilia Hernandez,^2^ Manohar Mutnal, PhD,^3^ Noah Henry,^3^ Katelyn Teckenbrock,^3^ Shikha Chaudhary, PhD,^3^ Paul D. Fey, PhD,^4^ John S. Stevenson, PhD,^4^ Yolande A. Chan, PhD,^4^ Alexander D. Pyden, MD, MPH, D(ABMM),^5^ Matthew Kearney, MHA, M(ASCP)^CM^,^5^ Benjamin von Bredow, PhD,^6^ Amer Ait Chebib,^6^ Elizabeth M. Garrett, PhD,^7^ Meng Pu, PhD,^7^ Shivaramu Keelara, PhD,^8^ Richard Remington,^8^ Cecilia Carvalhaes, MD, PhD, D(ABMM),^8^ Carrie Lasky, PhD,^8^ Theresa Okeyo Owuor, PhD,^8^ April M. Bobenchik, PhD^7*^

## **TABLE S1**

| **Table S1.** Antimicrobial susceptibility testing results for REVEAL as compared to MicroScan reported by organism**^a^** | | | | | | | | | | | | | | |
| --- | --- | --- | --- | --- | --- | --- | --- | --- | --- | --- | --- | --- | --- | --- |
|  |  |  |  | **MicroScan** | | | **VITEK REVEAL** | | | | | | | |
| **Organism** | **Microorganism**  **(n)** | **Antibiotic**  **(n)** | **Strains-**  **Antibiotics^b^** | **S** | **I** | **R** | **S** | **I** | **R** | **% EA (n)** | **% CA (n)** | **% VMD (n)** | **% MD (n)** | **% mD (n)** |
| *C. freundii* | 4 | 8 | 26 | 22 | 1 | 3 | 21 | 2 | 3 | 100 (26) | 96.2 (25) | 0.0 (0) | 0.0 (0) | 3.8 (1) |
| *C. koseri* | 4 | 9 | 35 | 35 | 0 | 0 | 35 | 0 | 0 | 100 (35) | 100 (35) | n/a (0) | 0.0(0) | 0.0 (0) |
| *E. cloacae* complex | 20 | 13 | 200 | 172 | 5 | 23 | 175 | 2 | 23 | 94.0 (188) | 92.5 (185) | 17.4 (4) | 2.3 (4) | 3.5 (7) |
| *E. coli* | 290 | 19 | 4752 | 4092 | 128 | 532 | 4073 | 137 | 542 | 98.1 (4660) | 95.6 (4544) | 3.6 (19) | 1.1 (44) | 3.1 (145) |
| *K. aerogenes* | 10 | 10 | 94 | 84 | 2 | 8 | 88 | 0 | 6 | 96.8 (91) | 94.7 (89) | 37.5 (3) | 0.0 (0) | 2.1 (2) |
| *K. oxytoca* | 20 | 13 | 233 | 220 | 2 | 11 | 218 | 2 | 13 | 98.3 (229) | 98.3 (229) | 9.1 (1) | 1.4 (3) | 0.0 (0) |
| *K. pneumoniae* group | 107 | 17 | 1688 | 1528 | 19 | 141 | 1523 | 24 | 141 | 99.2 (1675) | 97.7 (1649) | 3.5 (5) | 0.3 (5) | 1.7 (29) |
| *P. mirabilis* | 34 | 12 | 349 | 317 | 5 | 27 | 308 | 8 | 33 | 96.0 (335) | 94.3 (329) | 3.7 (1) | 2.5 (8) | 3.2 (11) |
| *S. marcescens* | 15 | 7 | 90 | 84 | 5 | 1 | 88 | 1 | 1 | 98.9 (89) | 95.6 (86) | 0.0 (0) | 0.0 (0) | 4.4 (4) |
| **Enterobacterales^c^** | **504** | **19** | **7467** | **6554** | **167** | **746** | **6529** | **176** | **762** | **98.1 (7328)** | **96.0 (7171)** | 4.4 (**33**) | 1.0 (**64**) | 2.7 (**199**) |
| *A. baumannii* | 4 | 4 | 13 | 13 | 0 | 0 | 12 | 1 | 0 | 92.3 (12) | 92.3 (12) | n/a (0) | 0.0 (0) | 7.7 (1) |
| *P. aeruginosa* | 45 | 11 | 465 | 427 | 12 | 26 | 438 | 6 | 21 | 97.4 (453) | 95.1 (442) | 19.2 (5) | 0.0 (0) | 3.9 (18) |
| **Overall** | **553** | **19** | **7945** | **6994** | **179** | **772** | **6979** | **183** | **783** | **98.1 (7793)** | **96.0 (7625)** | 4.9 (**38**) | 0.9 (**64**) | 2.7 (**218**) |
| **Abbreviations:** S, susceptible; I, indeterminate; R, resistant; EA, essential agreement; CA, categorical agreement; VMD, very major discrepant; MV, major discrepant; mD, minor discrepant | | | | | | | | | | | | | | |
|  | | | | | | | | | | | | | | |
| **^a^**Current VITEK REVEAL and MicroScan limitations are applied to the analysis  **^b^**Number for pair-wise comparisons  **^c^**Overall Enterobacterales | | | | | | | | | | | | | | |

## TA**BLE S2**

| **Table S2.** Performance comparison of VITEK REVEAL to MicroScan (reference test) for antimicrobial susceptibility testing | | | | | | | | | | | | | | | | | | | | | | | | | |  |
| --- | --- | --- | --- | --- | --- | --- | --- | --- | --- | --- | --- | --- | --- | --- | --- | --- | --- | --- | --- | --- | --- | --- | --- | --- | --- | --- |
|  |  | | | | **MicroScan** | | | | | | | | **VITEK REVEAL** | | | | | | | | | | | | |  |
| **Antimicrobial** | **Strains-Antimicrobials^a^** | | **S** | | | **I** | | **R** | | **S** | | **I** | | **R** | | **% EA** | | **% CA** | | **% VMD (n)** | | **% MD (n)** | | **% mD (n)** | |  |
| Amikacin**^b^** | | 407 | | 405 | | | 1 | | 1 | | 405 | | 1 | | 1 | | 100 (407/407) | | 100 (407/407) | | 0.0 (0) | | 0.0 (0) | | 0.0 (0) | |
| Amoxicillin-clavulanate**^c^** | | 415 | | 363 | | | 24 | | 28 | | 377 | | 7 | | 31 | | 98.6 (409/415) | | 93.0 (386/415) | | 10.7(3) | | 0.8 (3) | | 5.5 (23) | |
| Ampicillin-sulbactam | | 338 | | 208 | | | 69 | | 61 | | 205 | | 72 | | 61 | | 96.2 (325/338) | | 85.2 (288) | | 1.6(1) | | 2.9 (6) | | 12.7 (43) | |
| Aztreonam**^b^** | | 424 | | 333 | | | 7 | | 84 | | 338 | | 3 | | 83 | | 96.9 (411) | | 94.6 (401) | | 8.3 (7) | | 1.8 (6) | | 2.4 (10) | |
| Cefepime**^d^** | | 495 | | 406 | | | 4 | | 85 | | 405 | | 15 | | 75 | | 95.4 (472) | | 94.7 (469) | | 3.5 (3) | | 1.0 (4) | | 3.8 (19) | |
| Ceftazidime | | 453 | | 366 | | | 15 | | 72 | | 374 | | 1 | | 78 | | 96.9 (439) | | 93.8 (425) | | 6.9 (5) | | 1.9 (7) | | 3.5 (16) | |
| Ceftazidime-avibactam | | 392 | | 390 | | | 0 | | 2 | | 390 | | 0 | | 2 | | 100 (392) | | 100 (392) | | 0.0 (0) | | 0.0 (0) | | 0.0 (0) | |
| Ceftolozane-tazobactam | | 315 | | 305 | | | 2 | | 8 | | 304 | | 1 | | 10 | | 98.1 (309) | | 97.1 (306) | | 25.0 (2) | | 1.3 (4) | | 1.0 (3) | |
| Ceftriaxone | | 453 | | 370 | | | 5 | | 78 | | 368 | | 4 | | 81 | | 96.2 (436) | | 96.0 (435) | | 3.8 (3) | | 2.2 (8) | | 1.5 (7) | |
| Ciprofloxacin | | 447 | | 335 | | | 17 | | 95 | | 323 | | 21 | | 103 | | 97.5 (436) | | 92.8 (415) | | 3.2 (3) | | 2.1 (7) | | 4.9 (22) | |
| Ertapenem | | 348 | | 343 | | | 0 | | 5 | | 338 | | 3 | | 7 | | 99.4 (346) | | 98.6 (343) | | 0.0 (0) | | 0.6 (2) | | 0.9 (3) | |
| Gentamicin**^b,c^** | | 524 | | 468 | | | 4 | | 52 | | 469 | | 3 | | 52 | | 98.7 (517) | | 98.1 (514) | | 1.9 (1) | | 0.4 (2) | | 1.3 (7) | |
| Imipenem**^c^** | | 341 | | 330 | | | 8 | | 3 | | 333 | | 4 | | 4 | | 98.8 (337) | | 96.2 (328) | | 0.0 (0) | | 0.3 (1) | | 3.5 (12) | |
| Levofloxacin**^c^** | | 522 | | 415 | | | 7 | | 100 | | 406 | | 17 | | 99 | | 98.7 (515) | | 95.4 (498) | | 4.0 (4) | | 0.5 (2) | | 3.4 (18) | |
| Meropenem | | 512 | | 506 | | | 2 | | 4 | | 503 | | 2 | | 7 | | 99.6 (510) | | 99.2 (508) | | 0.0 (0) | | 0.4 (2) | | 0.4 (2) | |
| Meropenem-vaborbactam | | 327 | | 325 | | | 0 | | 2 | | 325 | | 0 | | 2 | | 100 (327) | | 100 (327) | | 0.0 (0) | | 0.0 (0) | | 0.0 (0) | |
| Piperacillin-tazobactam**^c,e^** | | 387 | | 359 | | | 7 | | 21 | | 353 | | 16 | | 18 | | 96.4 (373) | | 93.0 (360) | | 19.0 (4) | | 1.1 (4) | | 4.9 (19) | |
| Tobramycin**^c^** | | 527 | | 477 | | | 7 | | 43 | | 473 | | 13 | | 41 | | 97.7 (515) | | 96.6 (509) | | 0.0 (0) | | 0.8 (4) | | 2.7 (14) | |
| Trimethoprim-sulfamethoxazole**^c^** | | 318 | | 290 | | | 0 | | 28 | | 290 | | 0 | | 28 | | 99.7 (317) | | 98.7 (314) | | 7.1 (2) | | 0.7 (2) | | 0.0 (0) | |
| **Abbreviations:** S, susceptible; I, indeterminate; R, resistant; EA, essential agreement; CA, categorical agreement; VMD, very major discrepant; MV, major discrepant; mD, minor discrepant | | | | | | | | | | | | | | | | | | | | | | | | | |  |
|  | | | | | | | | | | | | | | | | | | | | | | | | | |  |
| **^a^**Number for pair-wise comparisons  **^b^**Antibiotics present in the GN BC-AST (RUO) panel used for this study but are not included on the GN02 commercial panel  **^c^**Results exclude reporting limitations for some organism/antibiotic combinations with specific MICs that require confirmatory testing with an alternate method as indicated in the IVD package insert  **^d^**For Enterobacterales, the susceptible-does dependent classification was used instead of intermediate  **^e^**For Enterobacterales, the either intermediate or susceptible-does dependent classifications were used, depending on the site; minimum inhibitory concentration was the same for both classifications | | | | | | | | | | | | | | | | | | | | | | | | | |  |

## **TABLE S3**

| **Table S3.** Performance of VITEK REVEAL compared to MicroScan across 553 gram-negative bacterial organisms from PBCs | | | | | |
| --- | --- | --- | --- | --- | --- |
| **Enterobacterales** | **EA** | **CA** | **VMD** | **MD** |  |
| Amikacin | 100 (363/363) | 100 (363/363) | 0.0 (0/1) | 0.0 (0/361) |  |
| Amoxicillin/Clavulanate | 98.6 (409/415) | 93.0 (386/415) | 10.7 (3/28) | 0.8 (3/363) |  |
| Ampicillin/Sulbactam | 96.2 (325/338) | 85.2 (288/338) | 1.6 (1/61) | 2.9 (6/208) |  |
| Aztreonam | 97.1 (368/379) | 95.8 (363/379) | 6.4 (5/78) | 2.0 (6/296) |  |
| Cefepime | 95.1 (428/450) | 94.4 (425/450) | 2.5 (2/80) | 1.1 (4/366) |  |
| Ceftazidime | 97.1 (436/449) | 94.0 (422/449) | 6.9 (5/72) | 1.9 (7/362) |  |
| Ceftazidime/Avibactam | 100 (350/350) | 100 (350/350) | 0.0 (0/2) | 0.0 (0/348) |  |
| Ceftolozane/Tazobactam | 97.8 (266/272) | 96.7 (263/272) | 25.0 (2/8) | 1.5 (4/262) |  |
| Ceftriaxone | 96.3 (436/453) | 96.0 (435/453) | 3.9 (3/78) | 2.2 (8/370) |  |
| Ciprofloxacin | 97.8 (395/404) | 93.3 (377/404) | 2.35 (2/89) | 2.3 (7/300) |  |
| Ertapenem | 99.4 (346/348) | 98.6 (343/348) | 0.0 (0/5) | 0.6 (2/343) |  |
| Gentamicin | 99.0 (476/481) | 98.3 (473/481) | 1.9 (1/52) | 0.5 (2/427) |  |
| Imipenem | 99.7 (308/309) | 96.7 (299/309) | 0.0 (0/2) | 0.3 (1/302) |  |
| Levofloxacin | 99.0 (472/477) | 96.0 (458/477) | 3.2 (3/93) | 0.5 (2/380) |  |
| Meropenem | 99.6 (461/463) | 99.1 (459/463) | 0.0 (0/3) | 0.4 (2/458) |  |
| Meropenem/Vaborbactam | 100 (327/327) | 100 (327/327) | 0.0 (0/2) | 0.0 (0/325) |  |
| Piperacillin/Tazobactam | 96.4 (373/387) | 93.0 (360/387) | 19.1 (4/21) | 1.1 (4/359) |  |
| Tobramycin | 97.5 (472/484) | 96.3 (466/484) | 0.0 (0/43) | 0.9 (4/434) |  |
| Trimethoprim/Sulfamethoxazole | 99.7 (317/318) | 98.7 (314/318) | 7.1 (2/28) | 0.7 (2/290) |  |
| **Subtotal** | 98.1 (7328/7467) | 96.0 (7171/7467) | 4.4 (33/746) | 1.0 (64/6554) |  |
|  |  |  |  |  |  |
| ***P. aeruginosa*** |  |  |  |  |  |
| Amikacin | 100 (40/40) | 100 (40/40) | 0.0 (0/0) | 0.0 (0/40) |  |
| Aztreonam | 95.6 (43/45) | 84.4 (38/45) | 33.3 (2/6) | 0.0 (0/37) |  |
| Cefepime | 97.8 (44/45) | 97.8 (44/45) | 20.0 (1/5) | 0.0 (0/40) |  |
| Ceftazidime/Avibactam | 100 (42/42) | 100 (42/42) | 0.0 (0/0) | 0.0 (0/42) |  |
| Ceftolozane/Tazobactam | 100 (43/43) | 100 (43/43) | 0.0 (0/0) | 0.0 (0/43) |  |
| Ciprofloxacin | 95.4 (41/43) | 88.4 (38/43) | 16.7 (1/6) | 0.0 (0/35) |  |
| Gentamicin | 95.4 (41/43) | 95.4 (41/43) | 0.0 (0/0) | 0.0 (0/41) |  |
| Imipenem | 90.3 (28/31) | 90.3 (28/31) | 0.0 (0/1) | 0.0 (0/27) |  |
| Levofloxacin | 95.6 (43/45) | 88.9 (40/45) | 14.3 (1/7) | 0.0 (0/35) |  |
| Meropenem | 100 (45/45) | 100 (45/45) | 0.0 (0/1) | 0.0 (0/44) |  |
| Tobramycin | 100 (43/43) | 100 (43/43) | 0.0 (0/0) | 0.0 (0/43) |  |
| Subtotal | 97.4 (453/465) | 95.2 (442/465) | 19.2 (5/26) | 0.0 (0/427) |  |
|  |  |  |  |  |  |
| ***A. baumannii*** complex |  |  |  |  |  |
| Amikacin | 100 (4/4) | 100 (4/4) | 0.0 (0/0) | 0.0 (0/4) |  |
| Ceftazidime | 75.0 (3/4) | 75.0 (3/4) | 0.0 (0/0) | 0.0 (0/4) |  |
| Imipenem | 100 (1/1) | 100 (1/1) | 0.0 (0/0) | 0.0 (0/1) |  |
| Meropenem | 100 (4/4) | 100 (4/4) | 0.0 (0/0) | 0.0 (0/4) |  |
| Subtotal | 92.3 (12/13) | 92.3 (12/13) | 0.0 (0/0) | 0.0 (0/13) |  |
| Overall | 98.1 (7,792/7,945) | 96.0% (7,625/7,945) | 4.9% (38/772)) | 0.9% (64/6,994) |  |
| **Abbreviations:** PBC, positive blood culture; EA, essential agreement; CA, categorical agreement; VMD, very major discrepancy; MD, major discrepancy | | | | | |

## **TABLE S4**

| **Table S4.** Agreement and error for VITEK REVEAL as compared to MicroScan for detection of ESBL species**^a^** | | |
| --- | --- | --- |
| **All species (n=393)** | **MicroScan** | |
| **VITEK REVEAL** | Positive | Negative |
| Positive | 63 | 17 |
| Negative | 2 | 311 |
| **Categorical Agreement** | 95.2% |  |
| **Very major discrepancy** | 3.1% |  |
| **Major discrepancy** | 5.2% |  |
|  |  |  |
| ***E. coli* (n=250)** | **MicroScan** |  |
| **VITEK REVEAL** | Positive | Negative |
| Positive | 50 | 8 |
| Negative | 2 | 190 |
| **Categorical Agreement** | 96.0% |  |
| **Very major discrepancy** | 3.8% |  |
| **Major discrepancy** | 4.0% |  |
|  |  |  |
| ***K. Pneumonia* (n=96)** | **MicroScan** |  |
| **VITEK REVEAL** | Positive | Negative |
| Positive | 11 | 4 |
| Negative | 0 | 81 |
| **Categorical Agreement** | 95.8% |  |
| **Very major discrepancy** | 0.0% |  |
| **Major discrepancy** | 4.7% |  |
| **^a^**Results represent data from 5 of the 6 study sites | | |

## **TABLE S5**

| **Table S5.** Overall antimicrobial susceptibility testing performance of VITEK REVEAL compared to MicroScan (reference) | |
| --- | --- |
| **Discrepancies** | **% (n/total)** |
| Very Major Discrepancy |  |
| Without BMD resolution | 4.9% (38/772) |
| With BMD resolution | 2.1% (16/772) |
| Major Discrepancy |  |
| Without BMD resolution | 0.9% (64/6994) |
| With BMD resolution | 0.7% (50/6994) |

| **Table S6.** Performance Characteristics of VITEK REVEAL Rapid AST Across Published Gram-Negative Blood Culture Studies | | | | | | | | | | | | | | |
| --- | --- | --- | --- | --- | --- | --- | --- | --- | --- | --- | --- | --- | --- | --- |
| **Study** | **Comparator** | **REVEAL Panel**  **used** | **# Sites** | **Country** | **Isolate**  **Number** | **CA Total**  **Number^a^** | **CA^b^** | **EA Total**  **Number** | **EA^c^** | **Number**  **Resistant** | **Very Major^d^** | **Number Susceptible** | **Major^e^** |  |
| Antonelli 2024(1) | BMD | GN01-AST | Single | Italy | 72 | 901 | 97.7%**^f^** | 895 | 97.9%**^f^** | CND | CND | CND | CND |  |
| Bianco 2024(2) | MicroScan**^g^** | GN01-AST | Single | Italy | 128 | 2220 | 97.6% | 2220 | 97.7% | 1081 | 1.6% | 1023 | 1.4% |  |
| Bui 2025a(3) | Disk diffusion | GN-BC AST RUO | Single | USA | 150 | 2309 | 94.8% | CND | CND | 279 | 4.3% | 278 | 2.9% |  |
| Bui 2025b(3) | BMD | GN-BC AST RUO | Single | USA | 150 | 2309 | 97.0% | CND | CND | 279 | 2.9% | 1957 | 0.3% |  |
| Calvo 2024(4) | BMD | GN01-AST | Single | Italy | 40 | 40 | 97.5% | 40 | 100% | 24 | 4.2%**^f^** | CND | CND |  |
| Couchot 2023(5) | Sensititre | Neg MDR MIC 1 | Single | France | 200 | 2200**^f^** | 96.1% | CND | CND | 375**^f^** | 1.6% | 1375**^f^** | 4.2% |  |
| Gitman et al (Current) | MicroScan | GN-BC AST RUO | Multi | USA | 553 | 7945 | 96.0% | 7945 | 98.1% | 772 | 4.9% | 6994 | 1.0% |  |
| Menchinelli 2024(6) | BMD | GN01-AST | Single | Italy | 50 | 1758 | 98.5%**^h^** | 1789 | 97.5%**^h^** | 712 | 1.0%**^h^** | 1020 | 0.3%**^h^** |  |
| Ostermann 2024(7) | MicroScan | Neg MDR MIC 1 | Single | Germany | 102 | 2002 | 97.1 | 1991 | 98.5 | 211 | 16.1% | 1678 | 0.5% |  |
| Park 2025a(8) | VITEK-2 | GN-BC AST RUO | Single | S. Korea | 107 | 824 | 94.5% | 854 | 97.9% | 195 | 0.5% | 648 | 0.6% |  |
| Park 2025b(8) | QMAC-dRAST**^i^** | GN-BC AST RUO | Single | S. Korea | 107 | 1129 | 94.1% | 1181 | 98.4 | 219 | 1.4% | 900 | 0.7% |  |
| Simmons-Williams 2026(9) | Accelerate Pheno**^j^** | GN BC-AST RUO | Single | USA | 128 | 1410 | 94.3% | 1410 | 96.0% | 228 | 7.5% | 1154 | 0.5% |  |
| Squitieri 2025(10) | BMD | GN01-AST | Single | Italy | 220 | 3603 | 98.3% | 3603 | 97.1% | 1036 | 1.1% | 2406 | 0.8% |  |
| Tibbetts 2022a(11) | VITEK-2**^k^** | Neg MIC 43 | Single | USA | 101 | 1615 | 96.2% | 1528 | 97.0% | 232 | 1.3% | 1342 | 0.3% |  |
| Tibbetts 2022b(11) | Sensititre**^l^** | Neg MIC 43 | Single | USA | 104 | 2258 | 96.3% | 2173 | 98.0% | 313 | 1.3% | 1889 | 2.6% |  |
| **Abbreviations:** AST, antimicrobial susceptibility testing; CA, categorical agreement; EA, essential agreement; BMD, broth microdilution; CND, could not determine; | | | | | | | | | | | | | | |
|  | | | | | | | | | | | | | | |
| **^a^**Represents total number of isolate/antibiotic combinations  **^b^**Categorical agreement = (number of isolate–antimicrobial results with matching S/I/R category ÷ total number of evaluable isolate–antimicrobial results) X100  **^c^**Essential agreement = (Number of isolate–antimicrobial MIC results within ±1 twofold dilution of comparator MIC ÷ total number of evaluable isolate–antimicrobial MIC comparisons) X100  **^d^**”Very major” indicates very major error or discrepancy = (False susceptible results ÷ total number of comparator-resistant results) X100  **^e^**^”^Major” indicates major error or discrepancy = (False resistant results ÷ total number of comparator-susceptible results) X100  **^f^**Group numbers and performance values were inferred from isolate numbers, number of antibiotics; and number of resistant and susceptible isolates reported in the article  **^g^**MicroScan (Beckman Coulter; USA)  **^h^**Performance values based on clinical and simulated isolates  **^i^**QMAC-dRAST (QuantaMatrix; South Korea)  **^j^**Accelerate Pheno (Accelerate Diagnostics, Inc; USA)  **^k^**VITEK-2 (BioMérieux; France)  **^l^**Sensititre (Thermo Fisher Scientific; USA) | | | | | | | | | | | | | | |

## **TABLE S6**

# **REFERENCES**

1.	Antonelli A, Cuffari S, Casciato B, Giani T, Rossolini GM. 2024. Evaluation of the Vitek(R) Reveal system for rapid antimicrobial susceptibility testing of Gram-negative pathogens, including ESBL, CRE and CRAB, from positive blood cultures. Diagn Microbiol Infect Dis 110:116503.

2.	Bianco G, Boattini M, Comini S, Bondi A, Curtoni A, Piccinini G, Musso T, Broccolo F, Cavallo R, Nordmann P, Costa C. 2024. Detection of volatile organic compounds as new paradigm to accelerate antimicrobial susceptibility testing: performance evaluation of VITEK(R) REVEAL. J Antimicrob Chemother 79:2237–2245.

3.	Bui TI, Brown AP, Muenks CE, Dumm RE. 2025. Comparison of VITEK REVEAL fast antimicrobial susceptibility testing to antibiotic disk diffusion for gram-negative bloodstream infections. J Clin Microbiol doi:10.1128/jcm.00927-25:e0092725.

4.	Calvo M, Maugeri G, Migliorisi G, Scalia G, Stefani S. 2024. The volatile organic compounds detection in MDR Gram-negatives antimicrobial susceptibility testing: Results from a four-month laboratory experience. Diagnostic Microbiology and Infectious Disease 110:116533.

5.	Couchot J, Fournier D, Bour M, Rajeev L, Rhodes P, Singh P, Jeannot K, Plésiat P. 2023. Evaluation of the Reveal® rapid AST system to assess the susceptibility of Pseudomonas aeruginosa from blood cultures. European Journal of Clinical Microbiology & Infectious Diseases 42:359–363.

6.	Menchinelli G, Squitieri D, Magri C, De Maio F, D'Inzeo T, Cacaci M, De Angelis G, Sanguinetti M, Posteraro B. 2024. Verification of the Vitek Reveal System for Direct Antimicrobial Susceptibility Testing in Gram-Negative Positive Blood Cultures. Antibiotics (Basel) 13.

7.	Ostermann G, Körber-Irrgang B, Krüger A, Singh P, Vo K, Gielen J, Aurbach U, Wisplinghoff H, Jazmati N. 2024. Performance evaluation of the Specific Reveal system for rapid antibiotic susceptibility testing from positive blood cultures containing Gram-negative pathogens. J Clin Microbiol 62:e0069224.

8.	Park J, Chung HS, Kim M, Bala Y, Hong G, Lee M, So MK. 2025. Clinical performance of the VITEK REVEAL fast antimicrobial susceptibility test within a real-world workflow for gram-negative bacteremia: comparison with QMAC-dRAST and conventional methods. Microbiol Spectr doi:10.1128/spectrum.01972-25:e0197225.

9.	Simmons-Williams C, Soares L, Becker D, Lima A, Rowe L, Uy D, Salazar H, Okeyo Owuor T, Keelara S, Remington R, Carvalhaes C, Silbert S. 2026. Comparison of the VITEK REVEAL AST and Accelerate Pheno systems for fast antimicrobial susceptibility testing of gram-negative blood cultures at a large academic health system. J Clin Microbiol 64:e0107325.

10.	Squitieri D, Menchinelli G, Magrì C, D'Inzeo T, Fiori B, Cacaci M, Sanguinetti M, De Angelis G, Posteraro B. 2025. Improving time-to-result: head-to-head comparison of three rapid AST systems for Gram-negative bacteremia, including the newly developed VITEK REVEAL. J Clin Microbiol 63:e0105025.

11.	Tibbetts R, George S, Burwell R, Rajeev L, Rhodes PA, Singh P, Samuel L. 2022. Performance of the Reveal Rapid Antibiotic Susceptibility Testing System on Gram-Negative Blood Cultures at a Large Urban Hospital. J Clin Microbiol 60:e0009822.
